# Dual Effects of Ag Doping and S Vacancies on H_2_ Detection Using SnS_2_-Based Photo-Induced Gas Sensor at Room Temperature

**DOI:** 10.3390/ma18122687

**Published:** 2025-06-06

**Authors:** Shaoling Wang, Xianju Shi, Na Fang, Haoran Ma, Jichao Wang

**Affiliations:** 1 School of Energy and Chemical Engineering, Puyang Vocational and Technical College, Puyang 457000, China; shaolingwang_518@sohu.com (S.W.); fangna9067@163.com (N.F.); 2 Puyang Institute of Technology, Henan University, Puyang 457000, China; 3 College of Chemistry and Chemical Engineering, Henan Institute of Science and Technology, Xinxiang 453003, China; mhrmhaoran@163.com

**Keywords:** SnS_2_, gas sensor, Ag doping, light-activated, H_2_ detection

## Abstract

Hydrogen (H_2_) monitoring demonstrates significant practical importance for safety assurance in industrial production and daily life, driving the demand for gas-sensing devices with enhanced performance and reduced power consumption. This study developed a room-temperature (RT) hydrogen-sensing platform utilizing two-dimensional (2D) Ag-doped SnS_2_ nanomaterials activated by light illumination. The Ag-SnS_2_ nanosheets, synthesized through hydrothermal methods, exhibited exceptional H_2_ detection capabilities under blue LED light activation. The synergistic interaction between silver dopants and photo-activation enabled remarkable gas sensitivity across a broad concentration range (5.0–2500 ppm), achieving rapid response/recovery times (4 s/18 s) at 2500 ppm under RT. Material characterization revealed that Ag doping induced S vacancies, enhancing oxygen adsorption, while simultaneously facilitating photo-induced hole transfer for surface hydrogen activation. The optimized sensor maintained good response stability after five-week ambient storage, demonstrating excellent operational durability. Experimental results further demonstrated that Ag dopants enhanced hydrogen adsorption–activation, while S vacancies improved the surface oxygen affinity. This work provides fundamental insights into defect engineering strategies for the development of optically modulated gas sensors, proposing a viable pathway for the construction of energy-efficient environmental monitoring systems.

## 1. Introduction

As a carbon-neutral energy carrier with a high gravimetric energy density, hydrogen has emerged as a pivotal renewable energy source for applications ranging from chemical synthesis to fuel cell technologies and industrial combustion systems [1,2]. However, the widespread adoption of hydrogen energy requires the urgent resolution of its inherent safety concerns, particularly given its hazardous nature, including its wide explosive concentration range (4.0–75.0 vol%), high diffusion coefficient (0.61 cm^2^/s), and substantial combustion enthalpy (285.5 kJ/mol) [3,4,5]. Thus, it is of great significance to selectively detect trace hydrogen [6]. These intrinsic properties underscore the critical need to develop reliable detection systems capable of selective trace-level hydrogen monitoring (particularly < 1000 ppm) in operational environments. The current hydrogen detection methodologies primarily employ analytical techniques such as gas chromatography (GC), semiconductor gas sensors, and optical spectroscopy approaches [1,6] In contrast, semiconductor-based chemiresistive sensors have garnered significant research interest due to their inherent advantages of low-cost fabrication, scalable manufacturing processes, and balanced performance metrics in terms of sensitivity, stability, and selectivity.

SnS_2_, as a 2D layered transition metal disulfide, is regarded as a promising gas-sensing material due to its large surface area and great electrical properties [7]. A series of SnS_2_-based materials have exhibited good gas sensitivity for NO_2_, NH_3_, xylene, C_2_H_5_OH, and H_2_S molecules [8,9,10,11,12]. W. Gao et al. demonstrated that a Pd/SnS_2_/SnO_2_ sensor exhibited a strong and rapid response to 500 ppm H_2_ at 300 °C [5]. However, to activate the gas detection abilities of semiconductors, a high operating temperature range of 100–450 °C is typically required, with an additional heater [13,14]. Considering its high resistance and weak adsorption to H_2_ molecules at room temperature (RT), the structural design of a SnS_2_ surface under external non-thermal field conditions would play a crucial role in solving the above issues [14,15]. Due to the excellent light absorption capacity of SnS_2_, light irradiation could be an effective alternative to thermal driving to activate its adsorption behavior toward gas molecules [15]. Under illumination, abundant electron–hole pairs could promote electron transfer, further improving the gas-sensing properties. While UV illumination (λ < 400 nm) has demonstrated effective photo-activation, its practical application is limited by photon-induced material degradation and potential health hazards. Recent advances in LED technology offer a safer alternative through visible light irradiation, combining sufficient photon energy for electron excitation with low power consumption [13]. Therefore, the synergistic combination of defect-engineered SnS_2_ nanostructures and LED photo-activation is of significance in developing an energy-efficient H_2_ sensor operating at RT.

In addition, the strategic introduction of sulfur vacancies (Vs) in SnS_2_ lattices enables selective oxygen chemisorption through isovalent orbital interactions, capitalizing on the chalcogen-group electronic affinity that facilitates superoxide radical (O_2_^−^) formation at defect sites. Simultaneously, noble metal dopants with high work functions have demonstrated superior hydrogen spillover effects, with their d-orbital electron configurations enabling optimized hydrogen binding energies through charge-transfer interactions [16,17]. In the intrinsic 2D SnS_2_ lattice, the Sn atom was sandwiched between two layers of S atoms maintained by van der Waals forces [18]. Doped Ag(I) impurities not only led to an adsorbed activated site for hydrogen but also facilitated the formation of S vacancies due to the charge and size differences between Ag(I) and Sn(IV) in SnS_2_ [18]. Thus, it would be of great interest to investigate the synergistic effects of S vacancies and Ag impurities on the sensing performance of Ag-doped SnS_2_ sensors.

In this regard, a series of Ag-doped SnS_2_ (Ag-SnS_2_) sheets was synthesized and used as sensing materials to sense H_2_ with the help of LED light illumination. The photo-activated Ag-SnS_2_ sheet showed significantly enhanced H_2_-sensing performance with a wide serviceable range from 5.0 ppm to 2500 ppm at RT. Such excellent gas sensitivity was attributed to the effects of Ag doping and light illumination. The influence of Ag doping on the surface structure was investigated by ESR and TEM. Additionally, through O_2_ absorption measurements and in situ XPS, the possible mechanism was proven.

## 2. Materials and Methods

The SnS_2_-based sample was synthesized through the one-step solvothermal method. All chemicals were directly obtained from Shanghai Macklin Biochemical Technology Co., Ltd. Initially, 2.5 mL Triton X-100, 5.0 mmol citric acid, and 5.0 mmol SnCl_4_·5H_2_O were dissolved in 30 mL of deionized water and then added to 0.15 mmol of AgNO_3_ under strong stirring conditions. Then, 5 mL thioacetamide solution (2.0 mmol/mL) was slowly added to the above colloidal solution under stirring. After ultrasonic processing for 30 min, the resulting mixture was transferred to a Teflon-lined autoclave and heated at 150 °C for 12 h. The Ag-SnS_2_ composite was finally obtained by centrifugation and washed with water/ethanol (three times), with subsequent drying at 70 °C. This sample was named xAg-SnS_2_, in which x indicates the ideal ratio (mol/mol %) between Ag and Sn elements. The pristine SnS_2_ sample was prepared using a similar method but without the addition of AgNO_3_. The SnS_2_-Vs sample, as a contrasting sample, was obtained from pristine SnS_2_ using 300 °C calcination under a 5%H_2_/95%N_2_ mixed atmosphere. The gas sensitivity was measured using reconstructive W30-A gas-sensitive equipment (Zhengzhou WeiSheng Electronics Technology Co., Ltd. Figures S1 and S2) [19,20]. The selectivity (Q) was defined as the ratio of SA/ST, where S_A_ and S_T_ denote the average sensing responses in the testing gas and the total testing gas, respectively [21,22]. The theoretical detection limit (LOD) of the 3Ag-SnS_2_ sensor was also calculated [23,24]. Thus, SnS_2_-based gas sensors were formed, and the synthesis process is detailed in Supplementary Materials S1. The morphological and structural characterization of the obtained SnS_2_ samples was performed through XRD, TEM, and XPS measurements. The S vacancies of doped SnS_2_ were explored using the ESR test(Bruker EMXplus). The above measurements are detailed in Supplementary Materials S2.

## 3. Results

**Figure 1:**
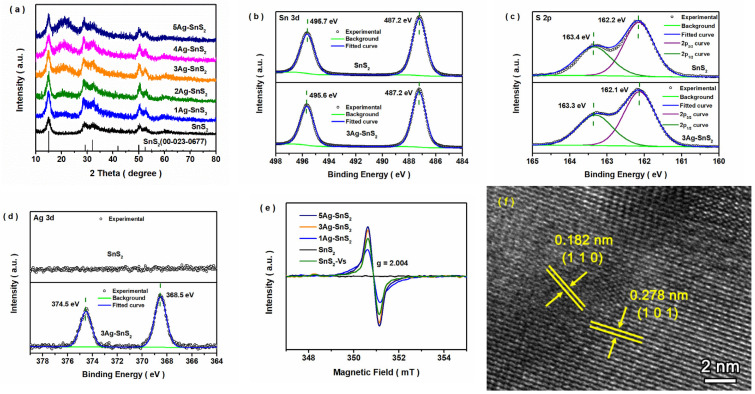
Structure, composition, defects, and morphology of SnS_2_ and doped SnS_2_ samples. (**a**) XRD pattern, (**b**) XPS: Sn 3d high-resolution spectra, (**c**) XPS: S 2p high-resolution spectra, (**d**) XPS: Ag 3d high-resolution spectra, (**e**) ESR of SnS_2_ and Ag-SnS_2_ samples, and (**f**) HR-TEM of 3Ag-SnS_2_ sample.

As shown in Figure 1, five obvious peaks at 15.0°, 28.2°, 32.1°, 50.0°, and 52.5° were found, corresponding to the characteristic peaks of the (0 0 1), (1 0 0), (1 0 1), (1 1 0), and (1 1 1) planes of hexagonal 2T SnS_2_ materials. With Ag addition, the intensity of one broad peak at about 22.0° gradually increased, while no new peak appeared. A broad peak between 20° and 30° appeared with Ag doping, and its intensity increased with increasing doped Ag content, indicating that the disordered atom part of Ag-SnS_2_ gradually increased [25,26]. Meanwhile, as shown in Figure S3, the characteristic peak of the (0 0 1) crystal facet moved toward a lower angle, which was due to the doped Ag impurities in the SnS_2_ structure [27,28]. Based on the ICP-OES results (Table S1), Ag impurities existed in the Ag-SnS_2_ sample, and its content increased with increasing Ag feedstock input. In the high-resolution Sn 3d spectra (Figure 1b), two stronger peaks at binding energies of 487.2 and 495.7 eV were associated with Sn 3d_5/2_ and 3d_3/2_ of Sn(IV) [25,29]. As displayed in Figure 1c, two peaks at 161.9 eV and 163.1 eV were found, corresponding to S 2p_3/2_ and 2p_1/2_, respectively (Figure 1c) [30,31]. In Figure 1d, two peaks at 368.0 and 374.1 eV are shown, which were ascribed to 3d_5/2_ and 3d_3/2_ of Ag(I) [18,25]. The peaks of both Sn and S shifted negatively after Ag doping, indicating a change in the surrounding electronegativity around the atoms. In the ESR spectra (Figure 1e), a clear sign of S vacancies (g = 2.004) appeared in the sample of Ag-doped SnS_2_, and its intensity was enhanced with increasing Ag input [18]. Combined with the XRD results, it was found that the ordered arrangement of Sn-S was destroyed by the doped Ag impurities, further forming S vacancies. As shown in Figure S4, a sheet morphology appeared in the SnS_2_ and 3Ag-SnS_2_ samples. In Figure 1f, the lattice fringes with spacing of 0.278 and 0.182 nm correspond to the (1 0 1) and (1 1 0) crystal facets of hexagonal 2T SnS_2_. Therefore, a series of Ag-doped SnS_2_ sheets with S vacancies were obtained through a one-step hydrothermal process.

**Figure 2:**
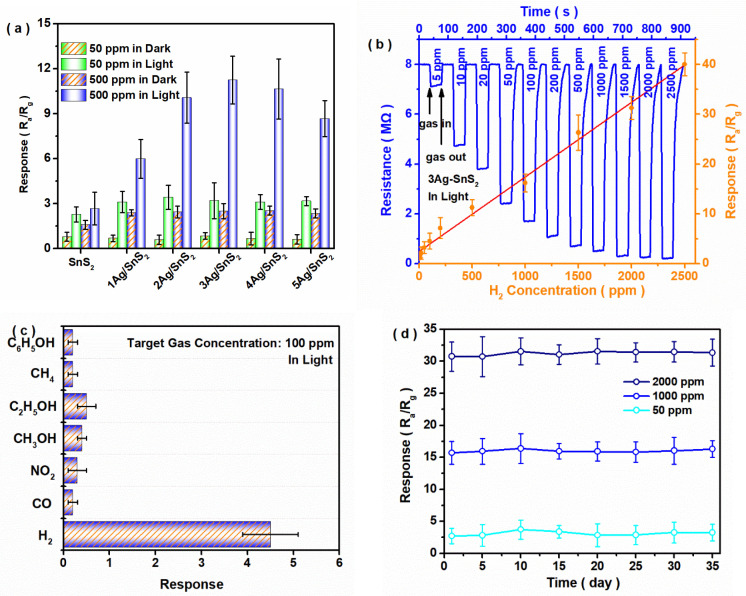
Performance of SnS_2_- and Ag-SnS_2_-based gas sensors. (**a**) Gas sensitivity for different samples; (**b**) resistance and response of 3Ag-SnS_2_ sensor at different H_2_ concentrations; (**c**) gas selectivity of 3Ag-SnS_2_ sensor for different samples; (**d**) cycling stability of 3Ag-SnS_2_ sensor over 5 weeks.

The gas sensitivity of the SnS_2_-based materials for H_2_ detection was investigated. As shown in Figure S6, the SnS_2_-based sensor at a 150 °C operating temperature in the dark exhibited higher gas sensitivity than that at a lower temperature. As shown in Figure 2a, all SnS_2_ materials exhibited sensitivity for H_2_ gas at room temperature, and the response under illumination was apparently higher than that in the dark. With increasing Ag addition, the response value of the Ag-SnS_2_ sensor was gradually increased, and the 3Ag-SnS_2_ sample showed the highest response value for H_2_ detection. Beyond the optimal value, its sign exhibited a marked decline, which was caused by the excess defects on the SnS_2_ surface, further decreasing the concentration of active electrons. As shown in Figure S6a, when faced with higher humidity, the gas sensitivity observably declined, especially for that under light. As shown in Figure S6b, smaller liquid contact angles appeared in all samples with S vacancies and/or Ag impurities, proving their good hydrophilicity at RT. This provides a possible reason for the weak water resistance: superfluous H_2_O molecules were readily adsorbed on the sensor’s surface, inhibiting the capture of H_2_ molecules. We further explored the relationship between the gas concentration and sensor sign. The results (Figure 2b) showed that, although a fast response rate for the 3Ag-SnS_2_ photo-induced sensor was found, its recovery time was gradually lengthened with increasing concentrations of H_2_ gas. Nonetheless, its response and recovery times under 2500 ppm H_2_ conditions were maintained at ~4 s and ~18 s, respectively. Moreover, a linear correlation (y = 0.01498x + 2.428, R^2^ 0.9923) could be determined at a H_2_ concentration between 5 ppm and 2500 ppm. The theoretical detection limit was calculated to be ∼923 ppb. Such a low LOD value satisfies the permissible exposure limits of H_2_ specified by various organizations. As shown in Figure 2c, when faced with multiple different target gases, the 3Ag-SnS_2_ sensor exhibited good selectivity for H_2_ gas. As summarized in Table S2, the Q value of the 3Ag-SnS_2_ sensor for H_2_ gas could reach 71.4%. The cycling stability of the gas sensor was also considered. As shown in Figure 2d, the photo-induced 3Ag-SnS_2_ sensor exhibited a stable response value for H_2_ detection at room temperature over 35 days. Compared with the previous SnS_2_- or SnO_2_-based gas sensors, as summarized in Table S3, the 3Ag-SnS_2_ photo-induced sensor exhibited good sensitivity, selectivity, and stability for H_2_ detection, with a wide concentration range (5.0 to 2500 ppm) when operating at room temperature [32,33,34,35,36,37,38,39,40,41].

**Figure 3:**
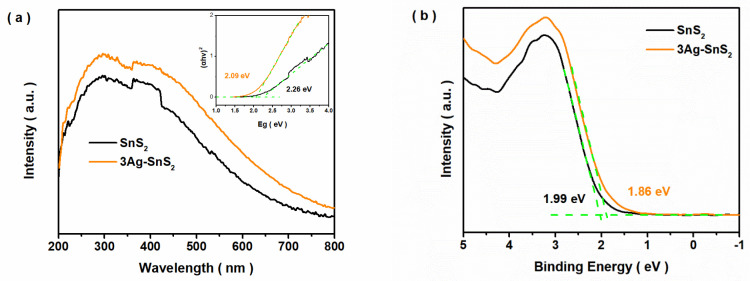
(**a**) DRS and (**b**) VB-XPS of SnS_2_ and 3Ag-SnS_2_ samples.

To explore the reasons for the enhanced photo-induced gas sensitivity of Ag-SnS_2_, the band structures and surface states of the obtained samples were further investigated. Firstly, the influence of Ag impurities on the band structure of SnS_2_ was studied. As shown in Figure 3a, the light response range of the 3Ag-SnS_2_ sample was wider than that of pristine SnS_2_, and their Eg values were calculated and are summarized in Table S4. Then, the VB band position could be measured through VB-XPS measurements. With Ag doping, the VB value modestly decreased, as shown in Figure 3b. Finally, the CB position could be calculated, and the information about their band structures is summarized in Table S4. Additionally, the photo-induced current curve is shown in Figure S7. Although the photo-induced current density of Ag-SnS_2_ was higher than that of SnS_2_, excess Ag impurities did not increase the abundance of photo-induced electrons. This may explain why S vacancies were easily formed around the doped Ag site due to the effects of charge balance; however, superfluous vacancies created a recombination center for photo-induced electron–holes, leading to a weak current density. This also explains why the 3Ag-SnS_2_ sample exhibited better gas sensitivity compared with other samples. As shown in Figure S8, the lift time of photo-induced electrons was calculated, and the average lift time of 3Ag-SnS_2_ (2.89 ns) was obviously longer than that of the pristine SnS_2_ sample (2.27 ns). Consistent with existing results about the photo-response current, doped Ag impurities could promote the separation of photo-induced carriers.

**Figure 4:**
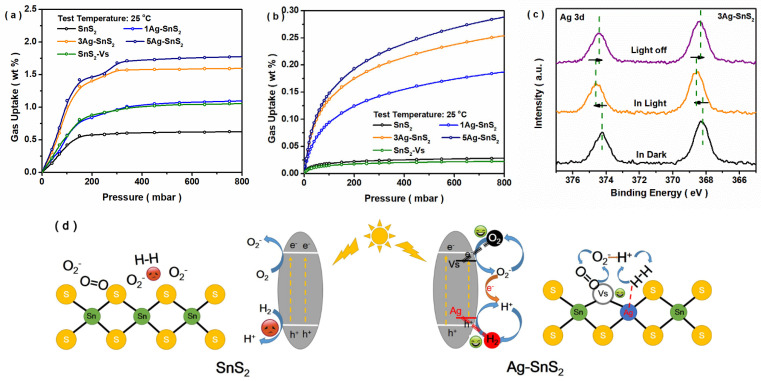
O_2_ (**a**) and H_2_ (**b**) absorption properties of SnS_2_ and Ag-SnS_2_ samples; in situ XPS results of Ag element in light/dark conditions; and (**c**) schematic diagram of gas sensing mechanism (**d**).

As shown in Figure 4a, all obtained samples showed adsorptive properties toward O_2_ gas at room temperature. It was found that the existence of Ag impurities enhanced the O_2_ adsorption capacity of SnS_2_, and its variation trend was in line with the change in S vacancies (Figure 1e). As shown in Figure 4b, the variation trend of the H_2_ adsorption property was similar to that of the O_2_ adsorption property. With the increasing density of Ag sites on the surface, the H_2_ adsorption capacity of SnS_2_ was increased, which was due to the superior hydrogen-binding capabilities of the high-work-function metal element. Meanwhile, the H_2_ adsorption property of SnS_2_-Vs was not increased through increasing that of O_2_ due to S vacancies. Hence, more adsorbed H_2_ molecules appeared around the Ag impurities. Additionally, the in situ XPS results (Figure 4c) showed that the peak positions of the Ag impurities in light shifted in a high-energy direction, and this trend was reversed after the light was switched off. It indicates that the electrons in Ag sites are lost under light, and this change in Ag atoms in light/dark conditions is reversible [42]. Hence, doped Ag sites may play a role in the surface transfer of photo-induced holes. Based on the above results, as shown in Figure 4c, the S vacancies around the Ag sites on the surface could easily adsorb the oxygen molecules. Then, metal Ag sites could capture hydrogen molecules in the air, which formed two neighboring adsorbed O_2_/H_2_ molecules. Simultaneously, under illumination, the photo-generated electrons and holes were transferred from SnS_2_ to the adsorbed O_2_ and H_2_ molecules, respectively. Moreover, superoxide radicals (O_2_^−^) and hydrogen ions (H^+^) were formed and reacted with each other, leading to electron consumption and a change in the resistance of SnS_2_. The possible mechanism behind the H_2_ detection of the photo-induced gas sensor is shown in Figure 4d.

## 4. Conclusions

In conclusion, a series of sheet-like Ag-doped SnS_2_ materials were prepared through the one-step hydrothermal method. S vacancies were generated, along with Ag impurity doping, leading to enhanced O_2_ adsorption properties. Compared to the pristine SnS_2_, which was unable to sense H_2_ at RT, the Ag-SnS_2_ sample exhibited excellent gas sensitivity for H_2_ detection, with a wide concentration range of 5.0 ppm to 2500 ppm under blue LED illumination. When faced with a high concentration of H_2_ gas (2500 ppm), its response and recovery times were maintained at ~4 s and ~18 s, respectively. Moreover, the sensor maintained its gas response after five weeks of relaxation. The synergistic effects of S vacancies and Ag impurities in enhancing O_2_/H_2_ adsorption have been proven. The Ag impurity sites played a key role in enabling photo-induced hole transfer to the active adsorbed hydrogen on the SnS_2_ surface under illumination. Given their remarkable gas-sensing performance, the formed Ag-doped SnS_2_ sheets could contribute to enhancing the potential and fueling the exploitation of next-generation, high-performance, gas-sensing, light-modulated devices at RT.

## Data Availability

The original contributions presented in this study are included in the article/Supplementary Material. Further inquiries can be directed to the corresponding authors.

## Supplementary Materials

The following supporting information can be downloaded at: https://www.mdpi.com/article/10.3390/ma18122687/s1, S1. Gas sensing characterization. S2. Sample characterization. Figure S1. Schematic diagram of photo-induced gas sensor. Figure S2. Wavelength range of LED light source. Figure S3. Partial XRD patterns of SnS_2_ and doped SnS_2_ samples. Figure S4. TEM images of SnS_2_ and 3Ag-SnS_2_ samples. Figure S5. Gas sensitivity of SnS_2_-based sensor at different operating temperatures in dark. Figure S6. Gas sensitivity of SnS_2_-based sensor at different humidity levels with 500 ppm H_2_ gas in light and liquid contact angle of water obtained over SnS_2_ sample surface. Figure S7. Photo-induced current curves of SnS_2_ and Ag-SnS_2_ samples. Figure S8. Transient photo-current curves of SnS_2_ and 3Ag-SnS_2_ samples. Table S1. Element content of obtained SnS_2_ sample according to ICP-OES. Table S2. Quantitative sensitivity values of 3Ag-SnS_2_ sensor. Table S3. Gas sensitivity of SnS_2_- or SnO_2_-based materials for H_2_ detection in previous studies and research. Table S4. Band structures of Ag-SnS_2_ and SnS_2_ materials.
