# Supplementary material for: Dual Effects of Ag Doping and S Vacancies on H2 Detection Using SnS2-Based Photo-Induced Gas Sensor at Room Temperature
Source: Materials (Basel). 2025 Jun 6;18(12):2687. doi: 10.3390/ma18122687 (PMC12193746; doi:10.3390/ma18122687)
Supplement: Supplementary file 1 [file materials-18-02687-s001.zip › materials-3636977-supplementary.pdf]

materials

MDPI

Supplementary Materials

Dual Effects of Ag Doping and S Vacancies on H_2 Detection Using SnS_2-Based Photo-Induced Gas Sensor at Room Temperature

Shaoling Wang [1,2], Xianju Shi [1,2,*], Na Fang [1,2], Haoran Ma [3] and Jichao Wang [3,*]

1 School of Energy and Chemical Engineering, Puyang Vocational and Technical College, Puyang 457000, China
2 Puyang Institute of Technology, Henan University, Puyang 457000, China
3 College of Chemistry and Chemical Engineering, Henan Institute of Science and Technology, Xinxiang 453003, China
* Correspondence: pyshixianju@163.com (X.S.); wangjichao@hist.edu.cn or wangjichao2016@163.com (J.W.)

S1. Gas sensing characterization

The gas sensing properties of SnS_2 based materials were characterized on a computer-controlled modified WS-30A gas sensor test system (Zhengzhou WeiSheng Electronics Technology Co.Ltd., China, Figure S1). The gas sensors were made of the alumina tube, on which two Au electrodes and platinum wires had been installed at each end. The sensors were tested under Blue LED (30W) and the light intensity on surface of gas sensor could reach 198~230 mW/cm^2 and its light wavelength spectra was showed in Figure S2.

The sensors were fabricated as follows: 15 mg of the SnS_2-based samples were mixed with 2 drops of terpineol to form a paste, which was then coated uniformly onto the surface of a ceramic tube with a pair of gold electrodes with a gap length of about 3 mm and connected by platinum wires. The obtained SnS_2 sensing film was vacuum dried at 80 ºC for 2 h. although a Ni-Cr resistor wire was put through the ceramic tube, in all test process, heating voltage still was closed. All the fabricated SnS_2 gas sensors were aged at RT for 3 days in air under Blue LED. Six parallel gas sensors were obtained for each powder sample, and the gas sensitivity was measured by two identical WS-30A systems to reduce the operating error. The size of sensing reactor reached 300mm × 300 mm × 200 mm, and the working volume without the inside test board was be calculated to be approximately 17.89 L. The side-heated gas sensors were made of alumina tube. The gas of tested target(high-purity level, >99.9%) was added around the air-fan using specialized gas injection through injection hole and its concentration was controlled by the added volume of tested gas. The liquid was added on the evaporator and the target gas was then generated in the sealed system to evaluate the response and selectivity in ambient atmosphere. When the environment temperature was set as 21.0~23.0 ºC, the ambient relative humidity (RH) was controlled by adding water on the evaporator. The target gases, including H_2, NO_2, CO, methyl alcohol, ethyl alcohol, methane and phenol, were introduced into the test system in various concentrations to assess the response and selectivity of the sensors at ambient atmosphere. The gas responses to reducing gases and to oxidizing gases were defined as $S = R_a/R_g$ and $S = R_g/R_a$, respectively, where R_a was the resistance of sensor in pure air, and R_g was the resistance of sensor in reducing or oxidizing gas. Short-term stability was evaluated by switching on/off from air to gas and back to air in different gas concentrations for a few cycles, whereas the long-term stability was investigated continuously for three

 https://doi.org/10.3390/ma18122687

months by repeating the short-term measurement under the condition of aging the device at RT.

S2. Sample characterization

Powder X-ray diffraction intensities were recorded with X-ray diffractometer (PANalytical X'Pert PRO, The Netherlands) operating at 40 kV and 40 mA, using CuK_{α} radiation ($\lambda_{K\alpha 1}$ = 1.5406 Å). The crystalline phases of SnS_2 calcined at different temperatures were analyzed with PANalytical X'Pert High Score Plus program and identified using the International Centre for Diffraction Data (ICDD) Powder Diffraction Files (PDF). In order to observe the morphology and characteristics of the sheet structure of SnS_2, transmission electron microscopy (TEM) was obtained on a Tecnai G^2 F20 S-TWIN electron microscope. Before measurements, the as-prepared samples were dispersed in ethanol and dripped onto a copper grid for the TEM tests. X-ray photoelectron spectroscopy (XPS) measurements were carried out at room temperature on a Perkin Elmer PHI 5300 X-ray Photoelectron Spectrometer. AlK_{α} radiation (hv = 1486.6 eV) was adopted as the excitation source, operating at 250W with 12.5 kV acceleration voltage. The binding energies (BE) were calibrated by using the background C1 s peak at 284.8 eV as internal reference. The deconvolution of the XPS peaks was performed using the XPS-PEAK 4.1 program, employing a Shirley background subtraction and Gaussian/Lorentzian symmetric line profiles for all the species. UV-vis diffuse reflectance spectra (DRS) were performed on a scan UV-vis spectrometer (Cary 5000) with integrating sphere detector. Photoelectrochemical tests in a conventional three-electrode system were recorded by a CHI 660E electrochemical workstation (Chenhua, Shanghai, China). The photocurrents of the obtained SnS_2 based samples were measured at 0.0 V (vs. Ag/AgCl) in Na_2SO_4 aqueous solution under UV-visible light with AM 1.5 filter after being purged by N_2 to remove O_2. The S vacancy of SnS_2 sample was investigated through electron spin resonance(ESR, Bruker EMXplus). In tested process, the Central magnetic field and The sweeping width was adopted to 3500.00 G and 150.00 G. The sweeping time and the microwave power were 30.00 s and 3.99 mW, respectively. the modulation amplitude and the conversion time set as 1.000 G and 40.0 ms. When the prepared SnS_2 based sample was subjected to a fixed strong magnetic field, the magnetic moments of the unpaired electrons interact with an applied electromagnetic wave. If the wave frequency meets the conditions of Equation (S1), electron spins resonate and absorb energy, as detected by an ESR device, resulting in an absorption peak.

$$h\upsilon = g\mu_0\beta H \quad \text{(S1)}$$

In this Equation, hν represents the energy of the electromagnetic wave, g is the spectral splitting factor, μ0 denotes the vacuum permeability, β is the Bohr magneton, and H stands for the magnetic field strength.

The gas responses as a function of H_2 concentration showed high linearity. The theoretical detection limit (LOD) of the 3Ag-SnS_2 can be calculated as follows:

$$LOD = 3 \times (RMS_{noise}/Slope) \quad \text{(S2)}$$

where LOD is the limit of detection, RMS_{noise} is the noise value for the sensor calculated using the variation in the relative sensor response in the baseline level using the root-mean-square deviation.

Table S1. Element content of obtained SnS_2 sample by ICP-OES.

Sample	Ag/Sn (mol/mol)		Sample	Ag/Sn (mol/mol)	
	ideal	tested		ideal	tested
SnS_2	-	-	1Ag-SnS_2	1:100	1.2:100
2Ag-SnS_2	2:100	1.9:100	3Ag-SnS_2	3:100	3.0:100
4Ag-SnS_2	4:100	3.9:100	5Ag-SnS_2	5:100	4.9:100

Table S2. Quantitative sensitivity values of the 3Ag-SnS_2 sensors.

Gas	Q value (%)	Gas	Q value (%)
H_2	71.4	CH_3CH_2OH	7.94
CO	3.17	CH_4	3.18
NO_2	4.76	C_6H_5OH	3.16
CH_3OH	6.35		

Table S3. Gas-sensitive property of SnS_2 or SnO_2 based materials for H_2 detection in previous studies and researches.

Materials	Gas-sensitive property	Ref.
SnO_2-Co_3O_4	Liner range: 5.0 ppm to 500 ppm<br>Operating temperature: 325 ℃<br>500 ppm: ~36.68	[1]
Au@SnO_2	Operating temperature: 450℃<br>200 ppm: 34.70	[2]
Sn_3O_4	Liner range: 3.0 ppm to 100 ppm<br>Operating temperature: 150 ℃<br>100 ppm: 4.70; 5.0 ppm: 1.90	[3]
In_2O_3/Al_2O_3-Pd/In_2O_3	Liner range: 1000 ppm to 10000 ppm<br>Operating temperature: 365 ℃<br>10000 ppm: ~92.00; 3000 ppm: 50.00	[4]
Pd/SnS_2/SnO_2	Liner range: 10 ppm to 100 ppm<br>Operating temperature: 300 ℃<br>100 ppm: ~12.05; 10 ppm: 1.65	[5]
Pd/SnO_2	Liner range: 2.0 ppm to 400 ppm<br>Operating temperature: 250 ℃<br>400 ppm: ~45.18; 2.0 ppm: ~3.17	[6]
single layer graphene	Liner range: 5.0 ppm to 200 ppm<br>Operating temperature: Room Temperature<br>265 nm UV light, 200 ppm: ~9.84; 5.0 ppm: ~1.86	[7]
Au/ZnO	Liner range: 50 ppm to 1000 ppm<br>Operating temperature: 250 ℃<br>365 nm UV light, 1000 ppm: 2.72; 50 ppm: 1.80	[8]
Pd/SnO_2	Liner range: -<br>Operating temperature: 160 ℃<br>100 ppm: 28.50	[9]
Pd–Au@SnO_2	Liner range: -<br>Operating temperature: 175 ℃<br>100 ppm: 28.50; 25 ppm: ~13.00	[10]
3Ag-SnS_2	Liner range: 5.0 ppm to 2500 ppm<br>Operating temperature: Room Temperature<br>Blue LED(30W), 2500 ppm: 40.02; 5.0 ppm: 1.12	This work

Table S4. Band structure of Ag-SnS_2 and SnS_2 materials.

Sample	E_{VB}	E_{CB}	Eg
SnS_2	1.99 V	-0.27 V	2.26 eV
3Ag-SnS_2	1.86 V	-0.23 V	2.09 eV

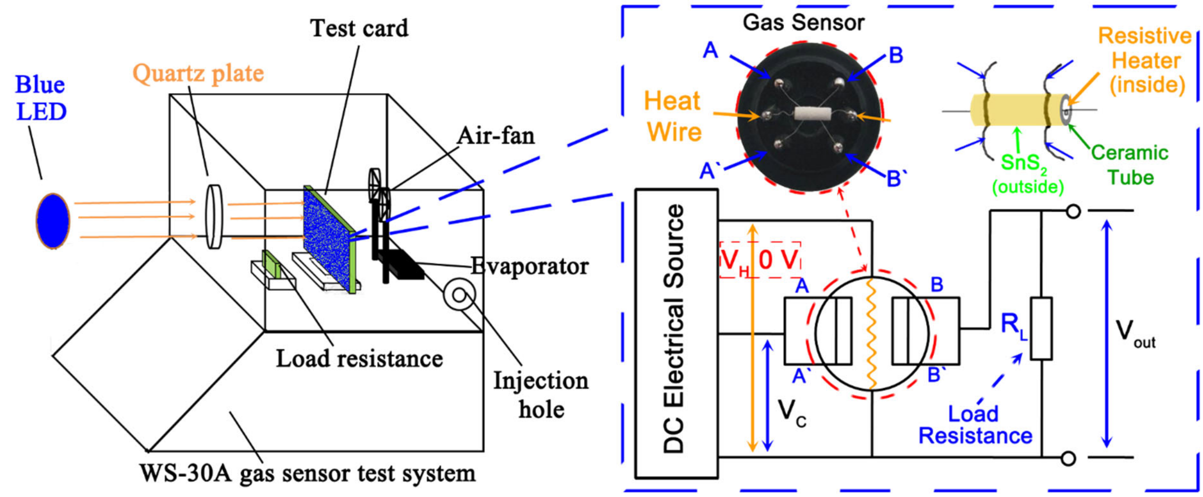

Figure S1. Schematic diagram of photoinduced-gas-sensor.

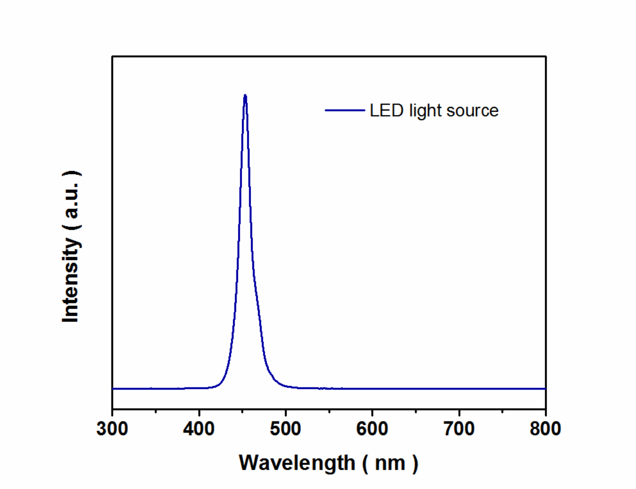

Figure S2. Wavelength range of LED light source.

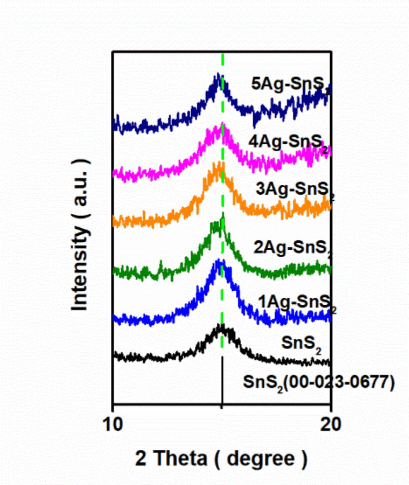

Figure S3. Partial XRD pattern of the SnS_2 and doped SnS_2 samples.

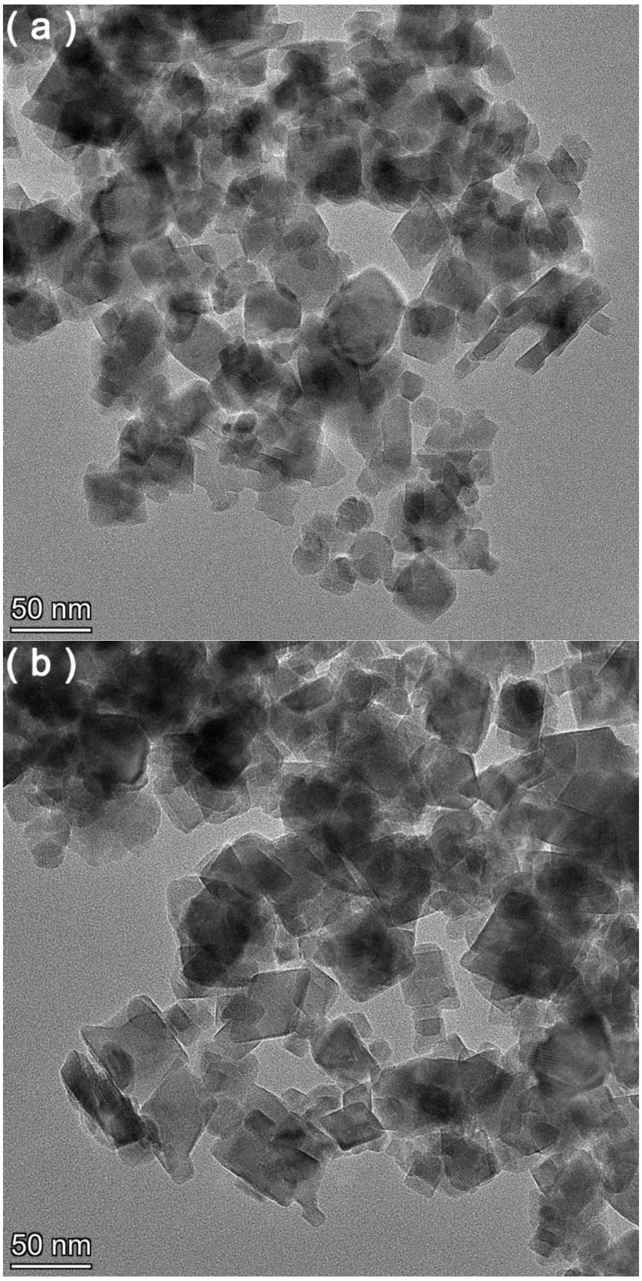

Figure S4. TEM images of SnS_2 (a) and 3Ag-SnS_2 (b) samples.

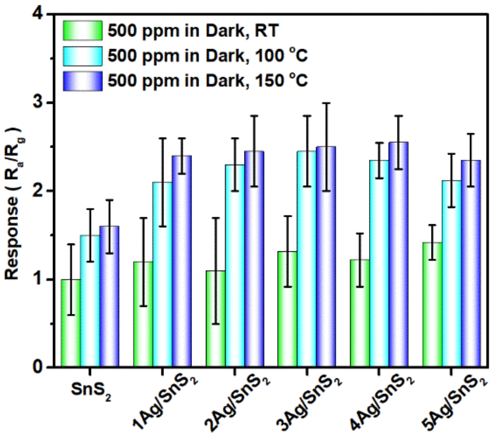

Figure S5. Gas-sensitive property of SnS_2 based sensor with different operating temperature in dark.

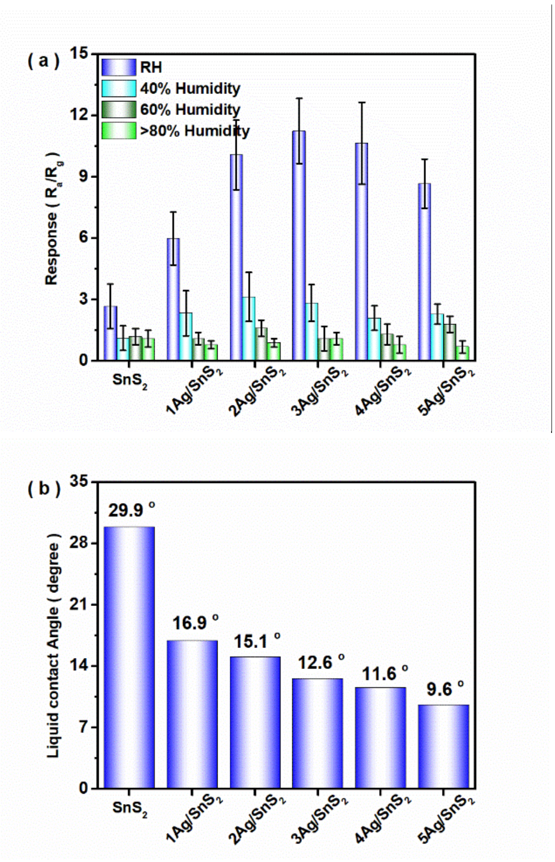

Figure S6. Gas-sensitive property of SnS_2 based sensor with different humidity for 500 ppm H_2 gas in light(a) and Liquid contact angle of water over obtained SnS_2 sample surface(b).

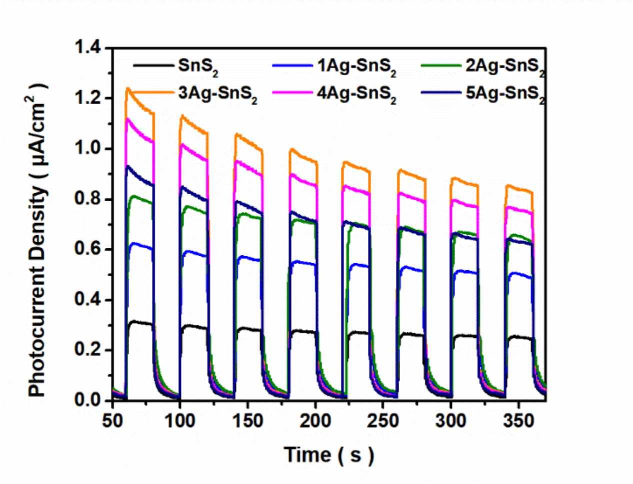

Figure S7. Photoinduced current curve of SnS_2 and Ag-SnS_2 samples.

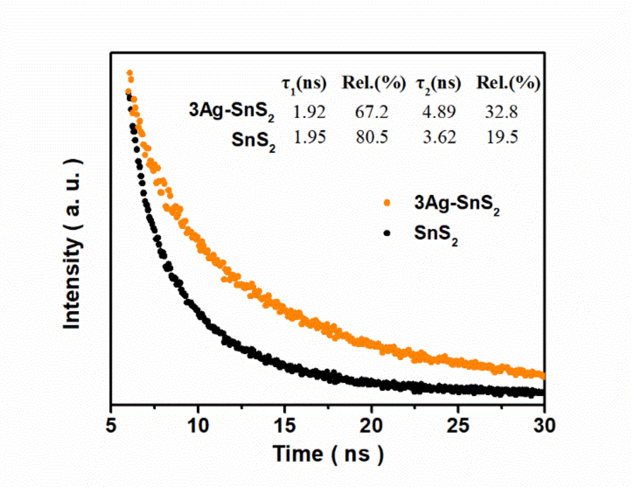

Figure S8. Transient photocurrent curves of SnS_2 and 3Ag-SnS_2 samples.

Reference

1. Qin, Y.; Zhang, Y.; Qiu, P.; Lei, S. SnO_2-Co_3O_4 nanocomposite sensor: Achieving ultra-selective hydrogen detection in mixed gas environments. *Sens. Actuators, B* **2025,** *422*, 136521. https://doi.org/10.1016/j.snb.2024.136521
2. Li, A.; Zhao, S.; Bai, J.; Xiao, H.; Gao, S.; Shen, Y.; Yuan, Z.; Meng, F. The role of AuSn alloys in optimizing SnO_2 nanospheres for chemoresistive hydrogen sensing. *Sens. Actuators, B* **2025,** *427*, 137214. https://doi.org/10.1016/j.snb.2024.137214
3. Liu, Y.; Chen, S.; Xiao, B.; Chu, J.; Wang, H.; Chen, Y.; Yao, T.; Yang, A.; Han, X.; Rong, M.; Wang, X. Ultra-large Sn_3O_4 nanosheets with Sn^{2+} defect for highly efficient hydrogen sensing. *Sens. Actuators, B* **2024,** *401*, 135025. https://doi.org/10.1016/j.snb.2023.135025
4. Liu, W.; Zou, J.; Li, S.; Li, J.; Li, F.; Zhan, Z.; Zhang, Y. Pd/In_2O_3-based bilayer H_2 sensor with high resistance to silicone toxicity and ultra-fast response. *Int. J. Hydrogen Energy* **2023,** *48*, 5743-5753. https://doi.org/10.1016/j.ijhydene.2022.11.043
5. Meng, X.; Bi, M.; Xiao, Q.; Gao, W. Ultrasensitive gas sensor based on Pd/SnS_2/SnO_2 nanocomposites for rapid detection of H_2. *Sens. Actuators, B* **2022,** *359*, 131612. https://doi.org/10.1016/j.snb.2022.131612
6. Lu, S.; Zhang, Y.; Liu, J.; Li, H.-Y.; Hu, Z.; Luo, X.; Gao, N.; Zhang, B.; Jiang, J.; Zhong, A.; Luo, J.; Liu, H. Sensitive H_2 gas sensors based on SnO_2 nanowires. *Sens. Actuators, B* **2021,** *345*, 130334. https://doi.org/10.1016/j.snb.2021.130334
7. Tang, C.; Jin, W.; Xiao, X.; Qi, X.; Ma, Y.; Ma, L. Graphene-based chemiresistive hydrogen sensor for room temperature operation. *Sens. Actuators, B* **2025,** *424*, 136889. https://doi.org/10.1016/j.snb.2024.136889
8. Kumar, G.; Li, X.; Du, Y.; Geng, Y.; Hong, X. UV-light enhanced high sensitive hydrogen (H_2) sensor based on spherical Au nanoparticles on ZnO nano-structured thin films. *J. Alloys Compd.* **2019,** *798*, 467-477. https://doi.org/10.1016/j.jallcom.2019.05.227
9. Wang, F.; Hu, K.; Liu, H.; Zhao, Q.; Wang, K.; Zhang, Y. Low temperature and fast response hydrogen gas sensor with Pd coated SnO_2 nanofiber rods. *Int. J. Hydrogen Energy* **2020,** *45*, 7234-7242. https://doi.org/10.1016/j.ijhydene.2019.12.152
10. Pandey, G.; Lawaniya, S. D.; Kumar, S.; Dwivedi, P. K.; Awasthi, K. A highly selective, efficient hydrogen gas sensor based on bimetallic (Pd–Au) alloy nanoparticle (NP)-decorated SnO_2 nanorods. *J. Mater. Chem. A* **2023,** *11*, 26687-26697. https://doi.org/10.1039/D3TA05878F

Disclaimer/Publisher's Note: The statements, opinions and data contained in all publications are solely those of the individual author(s) and contributor(s) and not of MDPI and/or the editor(s). MDPI and/or the editor(s) disclaim responsibility for any injury to people or property resulting from any ideas, methods, instructions or products referred to in the content.
